# System-specific periodicity in quantitative real-time polymerase chain reaction data questions threshold-based quantitation

**DOI:** 10.1038/srep38951

**Published:** 2016-12-13

**Authors:** Andrej-Nikolai Spiess, Stefan Rödiger, Michał Burdukiewicz, Thomas Volksdorf, Joel Tellinghuisen

**Affiliations:** 1 Department of Andrology, University Hospital Hamburg-Eppendorf, Hamburg, Germany; 2 Institute of Biotechnology, Brandenburg University of Technology, Cottbus - Senftenberg, Senftenberg, Germany; 3 Department of Genomics, Faculty of Biotechnology, University of Wroclaw, Wrocław, Poland; 4 Clinic and Polyclinic for Dermatology and Venerology, University Hospital Hamburg-Eppendorf, Hamburg, Germany; 5 Department of Chemistry, Vanderbilt University, Nashville, Tennessee 37235, USA

## Abstract

Real-time quantitative polymerase chain reaction (qPCR) data are found to display periodic patterns in the fluorescence intensity as a function of sample number for fixed cycle number. This behavior is seen for technical replicate datasets recorded on several different commercial instruments; it occurs in the baseline region and typically increases with increasing cycle number in the growth and plateau regions. Autocorrelation analysis reveals periodicities of 12 for 96-well systems and 24 for a 384-well system, indicating a correlation with block architecture. Passive dye experiments show that the effect may be from optical detector bias. Importantly, the signal periodicity manifests as periodicity in quantification cycle (*C*_*q*_) values when these are estimated by the widely applied fixed threshold approach, but not when scale-insensitive markers like first- and second-derivative maxima are used. Accordingly, any scale variability in the growth curves will lead to bias in constant-threshold-based *C*_*q*_s, making it mandatory that workers should either use scale-insensitive *C*_*q*_s or normalize their growth curves to constant amplitude before applying the constant threshold method.

Quantitative real-time polymerase chain reaction (qPCR) is the most widely applied molecular biology laboratory technique for gene expression analysis^1^. In addition to an accurate experimental design and a sensitive workflow^2^, qPCR data analysis constitutes a crucial step. To date, a plethora of algorithms has been developed for absolute and relative quantitation of qPCR data. The goal of these algorithms is the estimation of initial template fluorescence (*F*_0_) and copy number (*N*_0_). Most methods accomplish this in one of two ways: by estimating the amplification efficiency *E* as well as quantification cycle *C*_*q*_ and applying these two estimates to the basic exponential growth equation, or by fitting mechanistic models of PCR kinetics. In the former approaches, *E* is assumed constant up to *C*_*q*_ and is estimated by calibration curve analysis^3^ or from single curve fitting^4^,^5^,^6^,^7^,^8^. The mechanistic models avoid the calculation of *E* and *C*_*q*_ in directly estimating *F*_0_^9^,^10^,^11^, but they do so by tacitly setting *E* = 2 throughout the baseline region^12^.

The baseline region is commonly defined as the early cycles up to the point when the amplification curve rises detectably above the background fluorescence level. For reliable quantification, the raw fluorescence values *F*_*i*_ of the amplification curves need to be levelled to the *y*-axis origin, a process termed “baselining”. Common approaches are to calculate an averaged^15^,^16^,^17^, iteratively estimated^13^,^14^, or lower asymptote-derived value *F*_base_^5^,^6^,^8^ from the baseline region (e.g. *F*_1…10_) and then subtract this value from all fluorescence values prior to parameter estimation. Consequently, most of the published quantification algorithms conduct this data transformation^18^, along with other pre-processing steps such as smoothing or filtering^19^.

The above definition states the familiar display of signal *F*_*i*_ vs. cycle number *i* for a given reaction. However, with the widespread use of multi-reaction qPCR instruments, there is a second way to examine the fluorescence: by displaying the fluorescence values of all samples *k* at a fixed cycle *i*, e.g. *F*_10_ in sample 1, *F*_10_ in sample 2, …. *F*_10_ in sample *k*. This is exemplified in Fig. 1A and B: the black boxes denote *F*_10_, *F*_20_ and *F*_40_ for all 379 samples of a published technical replicate dataset.

We recently showed preliminary results^20^ for such an examination of a published large scale technical replicate dataset^18^ that revealed a pronounced regular and periodic pattern in the between-sample fluorescence signals for both early and late fixed cycle numbers. Subsequently, we have observed similar periodicity in many technical replicate datasets recorded with other qPCR platforms. Here, we employed autocorrelation analysis, a technique from the field of time series analysis that can reveal regularly occurring patterns in one-dimensional data. By this means, we have uncovered a periodicity of 24/12 in the qPCR raw data of 384/96-well microtiter plate systems that clearly corresponds to the plate architecture and/or optical read-out technology. This effect occurs at all cycle numbers and is typically stronger for cycles in the growth and plateau regions, so that the classical “baselining” fails to remove periodicities beyond the baseline region.

When *C*_*q*_ values are obtained using fixed threshold methods, the persisting plateau periodicity propagates exactly, hence resulting in periodic *C*_*q*_ values. However, this effect pertains to any systematic plateau phase scattering, periodic or not, making quantitation methods mandatory that are not directly influenced by the magnitude of the plateau phase. Interestingly, first- and second-derivative maxima methods^4^,^8^ are a viable choice because they are mathematically scale-independent and deliver random, non-periodic *C*_*q*_ values in the presence of periodic plateau phases. These findings lead to the simple conclusion to completely refrain from threshold-based qPCR quantitation.

To this end, we have developed a web application for users to examine their own qPCR data with respect to periodic and other non-random patterns.

## Results

### Periodicities in published and own technical replicate qPCR data

**Figure 1:**
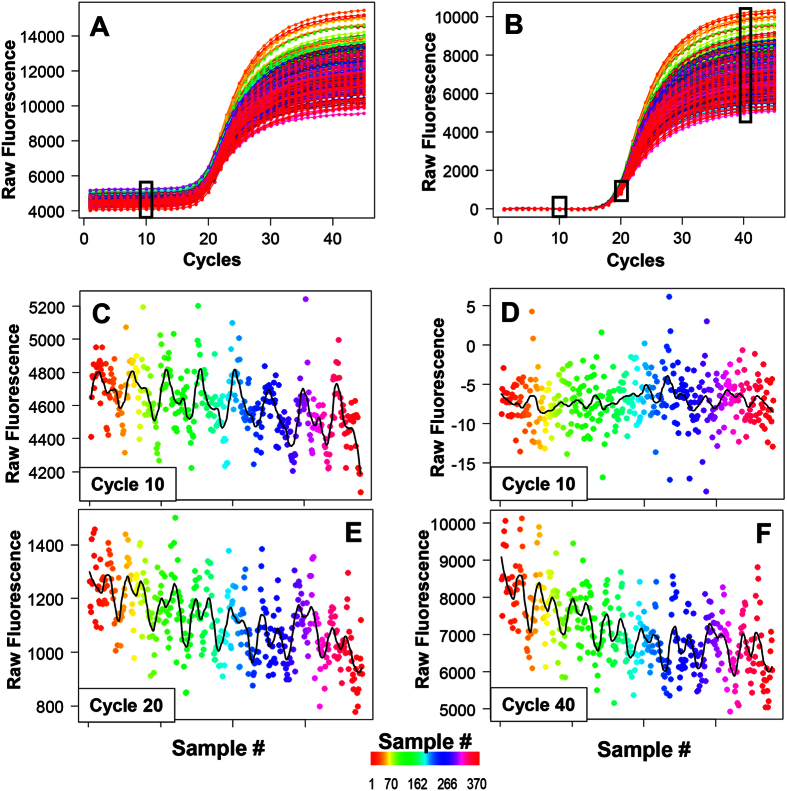
Discovering periodicity in raw and baselined qPCR data. **(A)** Plot of raw fluorescence values of the ‘380-replicates’ dataset from^18^, color-coded by sample number. **(B)** Same as in (**A**), but raw fluorescence data baselined by a linear model obtained from the first 10 cycles. Black boxes mark cycles 10, 20 and 40. (**C)** Fluorescence values at cycle 10 from the raw data of (**A**) throughout all 379 samples. A Loess smoother line was superimposed on the data to visualize periodicity. **(D),(E)** and **(F)** Fluorescence values at cycles 10, 20, and 40 from the baselined data of (**B**), with Loess smoother lines added in each case.

In a recent study^20^, we demonstrated periodic patterns in raw fluorescence values at cycles 1 and 45 of the ‘94-replicates-4-dilutions’ dataset^18^. A closer inspection of the ‘380-replicates’ dataset in Ruijter *et al*.^18^ revealed that the raw fluorescence values (Fig. 1A) are dispersed within a highly variable window of magnitudes (baseline region: 4000–5500, plateau region: 9000–15000). When *F*_10_ is plotted for all 379 samples, an added Loess smoothing line uncovers a clearly periodic pattern of the fluorescence values (Fig. 1C). After baselining the data with a linear model of *F*_1…10_ (Fig. 1B), the periodic pattern in *F*_10_ is completely removed and the fluorescence values exhibit a random-like pattern (Fig. 1D). However, for fluorescence values at later cycles, such as *F*_20_ in the exponential growth region (Fig. 1E) or *F*_40_ in the plateau region (Fig. 1F), the same periodic pattern is evident, showing that baselining does not compensate for intrinsic patterns beyond the baseline region. The same periodic pattern is present in each cycle of the data from the exponential phase onwards without exception. These observations indicate that there is periodic scale variability in the data, as otherwise baselining would correct the whole curve for periodicity.

**Figure 2:**
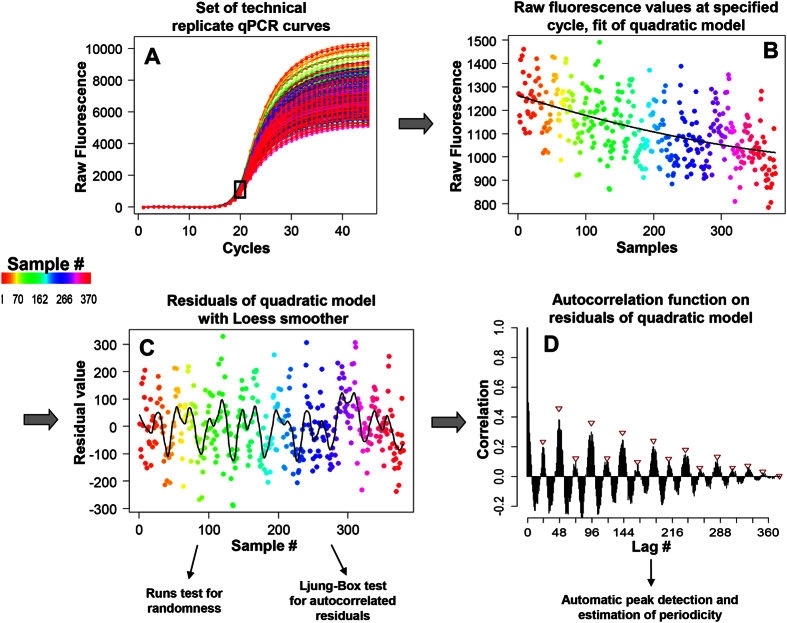
Principle analysis pipeline as demonstrated on the ‘380-replicates’ dataset. **(A)** Repetition of Fig. 1B, with selection of cycle 20 for periodicity analysis. **(B)** Cycle 20 data plotted against sample number and fitted to a quadratic model. **(C)** The residuals of the quadratic model are plotted against sample number, and a Loess curve is fitted to the data in order to visualize periodicity. A Runs test and a Ljung-Box test are performed to check for randomness and autocorrelated residuals, respectively. **(D)** The residuals from (**B**) are subjected to an autocorrelation function with lags 1…*n*, and the autocorrelations are plotted as a function of lag. Periodicities are estimated from the autocorrelation peaks by the *findpeaks* function.

**Figure 3:**
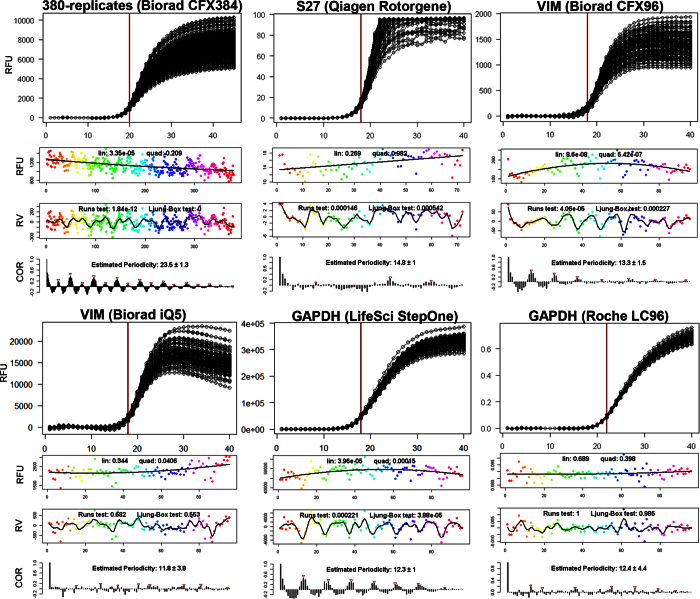
Detection of periodicity in baselined qPCR data acquired by six different hardware systems. Based on the analysis pipeline defined in Fig. 2, six different qPCR hardware systems (Bio-Rad CFX384, Biorad CFX96, Qiagen Rotorgene, Biorad iQ5, LifeSciences StepOne and Roche LC96) were analysed with respect to periodicity of fluorescence values for cycles early in the exponential region (red vertical line) using baselined data. Strong periodic patterns are evident for Biorad CFX384, Biorad CFX96 and LifeSciences StepOne while slight to almost negligible periodicity is visible for Qiagen Rotorgene, Biorad iQ5 and Roche LC96, as measured by Runs test and Ljung-Box test on the residuals. Omitted *x*-axis labels are those found for the different graphs types in Fig. 2. RFU: raw fluorescence units; RV: residual value; COR: autocorrelation.

To further investigate these findings on other qPCR systems, we generated five additional replicate datasets that differed in amplicon (VIM, GAPDH, S27), chemistry (SybrGreen I, EvaGreen), and qPCR instrument (CFX96, Rotorgene, iQ5, StepOne, LC96). We then used our developed analysis pipeline based on autocorrelation analysis (Fig. 2, see also Material & Methods) to uncover putative periodicities intrinsic to these datasets, including the ‘380-replicates’ dataset^18^. The latter, corresponding with the observations in Fig. 1, exhibits strong periodicity of ~24 that manifests in a distinct correlogram pattern (Fig. 3, top left). In this case, the Runs test for non-randomness and Ljung-Box test for autocorrelation are highly significant. Strong periodicities (Fig. 3) were also detectable for the ‘VIM CFX96’ and ‘GAPDH StepOne’ datasets, both with a period ~12–13, while ‘S27 Rotorgene’, ‘VIM iQ5’ and ‘GAPDH LC96’ displayed negligible systematic patterns (with insignificant non-randomness tests for the latter two).

The results from these five datasets suggest that strong periodicity is associated with some, but not all (i.e., not iQ5 or LC96) block-based systems.

### Propagation of plateau phase periodicities to *Cq* values

We next investigated the effect of periodic fluorescence on the estimation of threshold- (*C*_*t*_) and SDM (*C*_*q*SDM_)-based *Cq* values for the ‘380-replicates’ dataset. The rationale is that baselined periodic fluorescence *F*_*i,k*_ over all samples *k* at a fixed cycle *i* implies periodic threshold cycle values *C*_*t*_ at fixed threshold fluorescence *F*_*t*_ by propagation through the inverse function, from the following mathematical considerations:

Suppose a sigmoidal function such as a four-parameter sigmoidal model,


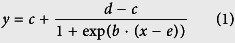


is fitted to qPCR data, and *C*_*t*_ values are estimated by the corresponding inverse function at threshold fluorescence *F*_*t*_,


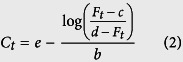


**Figure 4:**
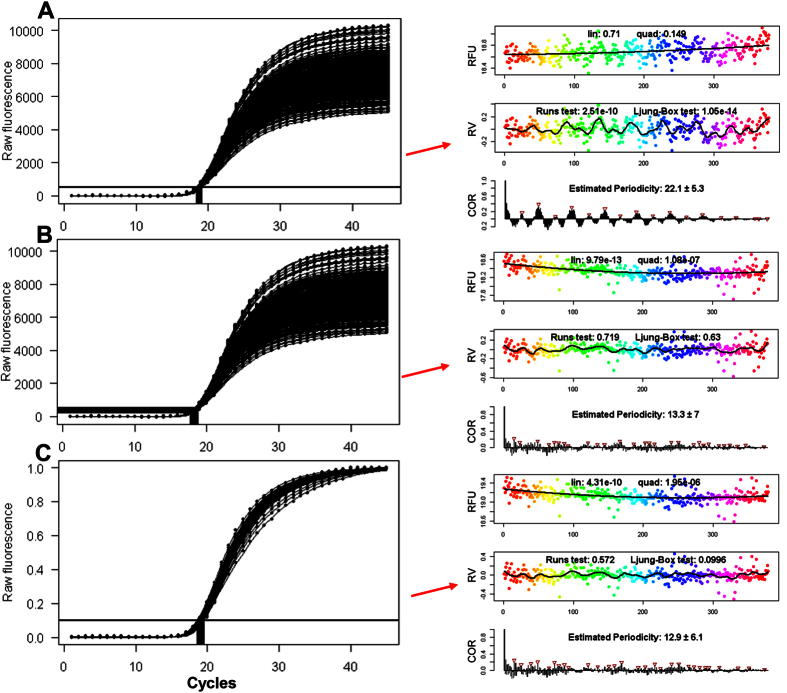
Impact of periodicity on *C*_*t*_ and *C*_*q*SDM_ estimation and the effect of normalization. **(A**) A five-parameter sigmoidal function was fitted to the baselined fluorescence values of the ‘380-replicates’ dataset and *C*_*t*_ values (red box) calculated at a threshold fluorescence of *F*_*t*_ = 500 by the inverse function. Autocorrelation analysis of these *C*_*t*_ values indicates strong and significant periodicity (right panel). **(B)** From the same fits as in (A), but *C*_*q*_ values estimated from SDM (*C*_*q*SDM_). Autocorrelation analysis of these *C*_*q*_ values indicates removal of periodicity and random pattern with insignificant Runs test *p*-value (right panel). **(C)** Fluorescence values were normalized (rescaled) into the interval [0, 1], fitted with a five-parameter sigmoidal model and *C*_*t*_ values calculated at *F*_*t*_ = 0.1 (red box). Similar to (**B**), autocorrelation analysis of the *C*_*t*_ values indicates removal of periodicity and random pattern with insignificant Runs test *p*-value (right panel). Omitted *x*-axis labels are those found for the different graphs types in Fig. 2. RFU: raw fluorescence units; RV: residual value; COR: autocorrelation.

Then, an increasing parameter *d* (upper asymptote) decreases *C*_*t*_ as the second term increases (*b* being negative, *e* being the first derivative maximum cycle). Indeed, estimated *C*_*t*_ values for the ‘380-replicates’ set at *F*_*t*_ = 500 (exponential region) exhibit strong periodicity (Fig. 4A) with exactly the same pattern as the fluorescence values for this dataset in Fig. 3.

As the overall scale of the qPCR curves drives the periodicity (it is strongest in the plateau phase, compare Fig. 1F), these results recommend the application of a scale-independent *C*_*q*_ marker to neutralize such effects. The SDM is a viable choice because of the following: a SDM-based *C*_*q*SDM_ value corresponds to the cycle number *x*, where the third derivative of (1),


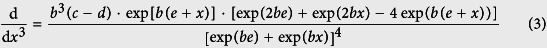


has the positive zero root


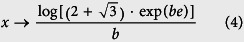


and is therefore scale-insensitive as both the parameters for lower asymptote *c* and upper asymptote *d* are cancelled out. The same accounts for the first-derivative maximum (not shown). Hence, *C*_*q*SDM_ is mathematically and physically decoupled from an overall periodic scaling of the fluorescence values. This paradigm is confirmed by the actual results: The *C*_*q*SDM_ values of the ‘380-replicates’ dataset are random and non-periodic (Fig. 4B; Runs test and Ljung-Box test are insignificant).

We then analysed a further technical replicate dataset consisting of seven 10-fold dilutions with 12 replicates each^34^, which was previously created with the widely used Lightcycler 480 system. The raw qPCR fluorescence values were fitted with a five-parameter sigmoidal model^8^,^24^, *C*_*t*_ values estimated, rescaled into the interval [0, 1] (as a consequence of the *C*_*t*_ value shifting in the dilution steps) and finally interrogated by autocorrelation analysis (Supplemental Fig. 3). Interestingly, a clear periodicity in the rescaled *C*_*t*_ values with a period of 12 is evident, demonstrating that periodic patterns can be extracted from replicate dilution series after rescaling and that this system delivers periodic data.

A potential alternative to using scale-insensitive *C*_*q*_ methods is to rescale all curves to the same final fluorescence magnitude before using threshold-based *C*_*t*_ estimation. This normalization approach was initially advocated by Larionov and coworkers^26^, who demonstrated improved standard curve regression statistics after such normalization. Indeed, normalizing the fluorescence values within the interval [0, 1] has the same effect as SDM-based estimation: all periodicity and non-randomness in *C*_*t*_ values is removed (Fig. 4C), although it appears that normalization does not completely remove intrinsic autocorrelation (Ljung-Box test *p*-value = 0.1). These results also pertain to all other datasets presenting periodicity (data not shown).

### *Cq* value periodicities acquired by published algorithms and vendor’s software

In a next step, we interrogated scale-sensitivity and periodicity in published data, where *C*_*t*_/*C*_*q*_ values are available from a variety of quantitation methods. We extended these considerations to the *C*_*q*_ estimation procedures of the methods compared in Ruijter *et al*.^18^ by similarly analysing the supplied *Cq, E* and *F*_0_ values for the ‘380-replicates’ data provided in their supplement. Specifically, we looked for putative periodicity in these parameters obtained from six different qPCR quantitation methods: LinRegPCR, FPKM, DART, FPLM, Miner, and 5PSM (Supplemental File 3). Four of these (LinRegPCR, FPKM, DART and FPLM) deliver periodic *C*_*q*_ values, while two (Miner, 5PSM) do not (Supplemental Fig. 1A). The estimated efficiencies exhibit no periodicity (Supplemental Fig. 1B), while the *F*_0_ values are periodic for LinRegPCR and FPLM (Supplemental Fig. 1C). The *F*_0_ values from the mechanistic MAK2 model display strong periodicity while the *C*_*q*_ values from the Cy0-method are random (Supplemental Fig. 1D+E). These observations clearly confirm that methods employing first- or second-derivative maxima (Miner, 5PSM, *Cy*_0_) yield non-periodic *C*_*q*_ values, which tallies with our results and mathematical derivations. It is also a logical consequence that *F*_0_ values estimated from *F*_0_ = *F*_*q*_/*E*^*Cq*^using periodic *C*_*q*_s and non-periodic *E*s are likewise periodic. In contrast, all *E*s are non-periodic, or only slightly periodic, when calculated by *E* = *F(C*_*t*_)/*F(C*_*t*_ − 1) and both nominator and denominator exhibit the same periodicity (albeit with a shift of one cycle). A potential cause for the lack of periodicity in SDM-based methods could be an increased dispersion (variance) of *C*_*q*_ values which obfuscates any periodic patterns. We therefore reanalysed the different *C*_*q*_ quantification methods from Ruijter *et al*.^18^ with respect to the dispersion of their calculated *C*_*q*_ values (Supplemental Fig. 1F). We found that the three SDM-based methods (*Cy*_0_, Miner, 5PSM) deliver *C*_*q*_ values with lower dispersion, which manifests in narrower boxplot boxes (in blue) and lower coefficients of variation (c.v., in blue). These results are not surprising as they constitute a re-evaluation of similar *C*_*q*_ dispersion results (compare Figure 6B in Ruijter *et al*.^18^) and tally with the observations from a replicate dilution set (compare Fig. 3 in Tellinghuisen & Spiess^20^). Moreover, they should largely be a consequence of the already mentioned decreased sensitivity of these three SDM-based methods to the overall plateau phase scattering.

As the above data were fitted with author-developed algorithms, we inspected whether *Cq* values also exhibit periodicity when obtained from the actual output of qPCR system analysis software. Indeed, using the ‘VIM.CFX96’ data to calculate *Cq* values by the CFX Manager™ software, we observed highly periodic *Cq* values from both supplied quantitation methods, “Manual threshold” and “Nonlinear regression” (Supplemental Fig. 2).

### Effect of periodic *Cq* values on calibration curve-derived efficiency and copy number estimation

The presence of periodic *Cq* values is likely to entail a quantification bias that depends on the location of the selected *Cq* values within the periodic pattern. To address the question on how large this selection bias can be, we conducted an iterative analysis on the ‘94-replicates-4-dilutions’ dataset^18^. We previously demonstrated that this dataset also exhibits extensive periodicity in fluorescence values^20^. Similar to the ‘380-replicates’ dataset analysed in this work, a fixed threshold estimation of the lowest dilution set (15000 copies) at *F*_*t*_ = 500 results in 94 periodic *C*_*t*_ values (Supplemental Fig. 4A). Using all combinations of the two most extreme *C*_*t*_ values of the lowest (15000 copies) and highest (15 copies) dilution as well as all 94 *C*_*t*_ values of the two intermediate dilutions (1500 and 150 copies), we created 34968 linear regressions for calibration-based absolute quantitation (Supplemental Fig. 4B). Efficiencies calculated from the slopes of the regression curves were spread within a window of 1.79 to 2.19 (Supplemental Fig. 4C), while copy numbers estimated at *C*_*t*_ = 30 varied from 28 to 86 (Supplemental Fig. 4D). These findings demonstrate that i) efficiency estimation is highly dependent on the combination of *C*_*t*_ values used for constructing the regression curve and ii) estimated copy numbers for unknowns must be viewed with caution as they can spread over a large interval.

### Factors contributing to qPCR periodicities

**Figure 5:**
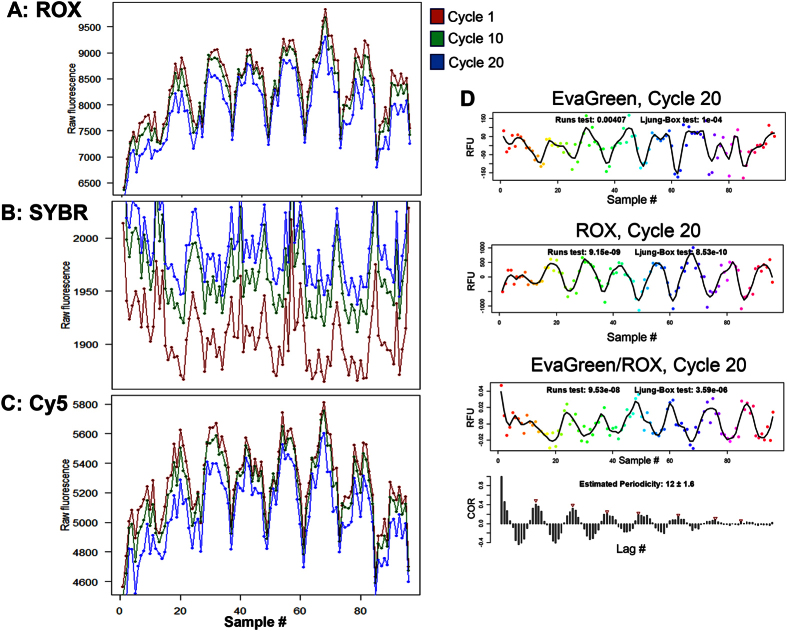
Non-amplification periodicity acquired by cycling a qPCR mastermix without template with three different fluorescent dyes. ROX **(A)**, SYBR Green **(B)** and a Cy5-labeled Oligo-dT_20_ oligonucleotide **(C)** were subjected to 40 cycles. Shown are the fluorescence values at cycles 1 (red), 10 (green) and 20 (blue) throughout all 96 samples, obtained from the three different channels. Periodicity is evident for ROX and Cy5, and to a lesser extent for SYBR Green. **(D)** The EvaGreen-based fluorescence values at Cycle 20 (with periodicity) was normalized with the corresponding ROX-based fluorescence values (with periodicity), resulting in periodic data with significantly lower magnitude. RFU: raw fluorescence units; COR: autocorrelation.

In principle, at least three factors could contribute to such periodicity effects: i) uneven thermal distribution of the Peltier block system, resulting in well-to-well differences in *E*, which in turn influence the amount of amplicon formation, ii) bias and heterogeneity of the optical detection system and iii) pipetting induced patterns, e.g. from uneven and tip-dependent deposition with multichannel pipettes. The first of these cannot account for the observed periodicity in the baseline fluorescence, where there is negligible signal from the amplicons. To address sources ii) and iii), we performed a simple experiment in which qPCR mastermixes without template, but containing SybrGreen, ROX or 150 nM Oligo-dT_20_-Cy5, were cycled and scanned in the corresponding channels (Fig. 5). The deposition of the mastermix in all 96 wells was conducted with a single-channel pipettor in order to avoid any periodic volume differences (source iii). We selected the CFX96 (Biorad) system because of its strong periodicity in fluorescence values during qPCR amplification (Fig. 3). Even in the absence of amplification, ROX (Fig. 5A), Cy5 (Fig. 5C) and to a lesser extent SYBR Green (Fig. 5B), displayed for cycles 1, 10 and 20 periodic fluorescence patterns that were highly similar. These results support source ii) - optical detection effects - as the primary source of the periodicity, consistent with the same being responsible for overall scale variability in the amplification profiles.

## Discussion

qPCR amplification profiles commonly display significant variability in their intensity scale. Using autocorrelation analysis, we have shown that for many block-based instruments, such effects are periodic in the sample number even after baselining, leading to similar periodicity in threshold-based estimates of the quantification cycle *C*_*q*_. The observed periodicities of ~12 for 96-well block systems and ~24 for a 384-well system suggest a correlation with block architecture (number of columns). Our passive dye experiments indicate that this effect is very likely due to optical detector bias, however positional block temperature effects on dye fluorescence magnitude may also play a role^33^. Due to fluorescence periodicity in the absence of any DNA template, we rule out possible influences on qPCR amplification efficiency, as proposed for positional bias in *C*_*q*_ and melting-curve-derived *T*_*m*_ values^21^,^22^,^29^. The periodicity of ROX fluorescence suggests - as is a widely applied procedure - to normalize SybrGreen fluorescence by ROX fluorescence through *F*_*i,k*_(SYBR)/*F*_*i,k*_(ROX). Conducting this approach for the ‘VIM.CFX96’ data with the corresponding ROX fluorescence at Cycle 20 certainly decreases the magnitude of observed periodicities (Fig. 5D, lower panel) from a range of [−100, 100] to [−0.02, 0.02], however the periodicity as such persists. In addition, we have observed a decrease in ROX fluorescence during cycling (Supplemental File 4), which may pose a problem for this approach. At this point it must be emphasized that we discovered highly periodic *Cq* values obtained from hardware systems that state to have an optical detection system which eliminates the need to use passive reference dyes, such as the CFX96 (Biorad) system (compare vendor’s info^32^). Finally, positional differences in qPCR efficiency would likely result in position-dependent amplicon yield. In another experiment (data not shown), we found no correlation between *F*_max_ and amplicon yield (as obtained from capillary electrophoresis), similar to other’s observations^26^. While heterogeneity in the robotic pipetting systems might also play a role^27^,^28^, this cannot explain the periodicity when we used a single-channel pipette to charge the wells. A most plausible explanation may be found in the different optical architectures of the qPCR systems. However, to this end, we do not feel entitled to give an undisputed explanation on which optical factors (e.g. spherical aberration of the lens/mirror system or “optical vignetting” in the field-of-view periphery) drive periodicity. For instance, block-based systems that did *not* show such effects (iQ5 and LC96) conduct simultaneous optical measurements of all samples (CCD camera and per-well fibre optics, respectively), instead of acquiring signals through a column-wise scanning optical shuttle (CFX384, CFX96, StepOne). On the other hand, another CCD camera/mirror system (LC480) clearly exhibited periodicity, such that a fixed scanning architecture is not necessarily devoid of delivering periodic fluorescence readouts.

These detection bias effects are clearly undesirable, and it must be recognized that their effects on *C*_*q*_ can be completely eliminated by using a scale-insensitive definition for *C*_*q*_ (*e.g.*, SDM, Cy0, relative threshold) or by normalizing the data to constant scale^26^. For the latter case, it is necessary to record data well into the plateau region, or to use a whole-curve fitting method that reliably estimates the plateau. Many vendors include an SDM option for *C*_*q*_ in their software, but the virtues of this and other scale-insensitive *C*_*q*_ markers over *C*_*t*_ have been underappreciated. It is further worth noting that the first two approaches also neutralize effects of true variability in the amplicon yield from random cycle-to-cycle variation in the amplification efficiency^30^. Most importantly, the observed periodicity, while interesting in itself, is merely an indicator that any kind of scale variability, periodic or random, propagates into threshold-based *C*_*t*_ values. Consequently, the widespread application of fixed threshold-based quantitation is highly questionable, although it is established as the most commonly used qPCR quantification method. We see various reasons for this: a) it was the first method introduced and implemented in vendors’ software during the dawn of qPCR technology, so that scientists might perceive it as robust and well-tested, b) it seems more intuitive and familiar to base the analysis on a single fixed parameter, similar to other analytic methods, c) unawareness of scale effects on fixed location indices in a sigmoidal curve, and d) lack of implementation in some qPCR software.

We advise researchers to use our approach to examine their qPCR data for periodicity, as this problem appears to be unacknowledged by the instrument vendors. To the best of our knowledge, none of the existing and previously reviewed qPCR software^31^ can identify periodic patterns in qPCR data. Our web application, www.smorfland.uni.wroc.pl/shiny/period_app/, fills this gap, making it easy for users to examine their data for periodic and other non-random patterns (more details in Supplemental Fig. 5).

## Materials and Methods

### Datasets

For the analysis of periodicity in this work, we have employed one published 384-reaction technical replicate dataset (‘380-replicates’^18^) and five new 72 to 96-reaction technical replicate datasets, obtained with different amplicons, qPCR hardware systems, and detection chemistries^23^. The parameters were as follows for the new datasets (gene; forward primer; reverse primer; qPCR instrument; detection chemistry; cycling parameters; primer concentration; amplification chemistry):

‘S27’: Ribosomal protein S27; aacatgcctctcgcaaagga; tgtgcatggctaaagaccgt; Qiagen Rotorgene; SybrGreen I; 95 °C 2′ = > (95 °C 10”, 60 °C 20”, 72 °C 30”) × 40, 0.2 μM, Takara ExTaq.

‘VIM.CFX96’: human Vimentin; cccttgacattgagattgcc; ccagattagtttccctcaggt; Biorad CFX96; EvaGreen; 95 °C 10′ = > (95 °C 30”, 59 °C 45”, 68 °C 45”) × 40, 0.2 μM, LifeTechnologies Maxima qPCR Kit.

‘VIM.iQ5’: human Vimentin; cccttgacattgagattgcc; ccagattagtttccctcaggt; Biorad iQ5; EvaGreen; 95 °C 10′ => (95 °C 30”, 59 °C 45”, 68 °C 45”) × 40, 0.2 μM, LifeTechnologies Maxima qPCR Kit.

‘GAPDH.StepOne’: Glycerinaldehyd-3-phosphat-Dehydrogenase; Assay Hs02758991_g1; LifeTechnologies StepOne Plus; TaqMan; 50 °C 2′, 95 °C 10′ => (95 °C 15”, 60 °C 60”) × 40; primer concentrations proprietary; Thermo Scientific Maxima Probe qPCR Master Mix.

‘GAPDH.LC96’: Glycerinaldehyd-3-phosphat-Dehydrogenase; Assay Hs02758991_g1; Roche Lightcycler 96; TaqMan; 50 °C 2′, 95 °C 10′ = > (95 °C 15”, 60 °C 60”) × 40; primer concentrations proprietary; Thermo Scientific Maxima Probe qPCR Master Mix.

Raw fluorescence data for these datasets are supplied in Supplemental File 1. All amplicons have been size-checked by capillary gel electrophoresis (Bioanalyzer, Agilent).

### Data transformations

When indicated, curves were fitted either with a five-parameter asymmetric model^8^ or with an interpolating cubic spline (details below). Baseline subtraction prior to fitting was conducted using a linear model of the form *F*_*cor.i*_ = *F*_*i*_ − (*ax*_*i*_ + *b*), with *a* and *b* obtained from linear regression of the first ten cycles. Normalisation to [0, 1] was performed by transformation with {*F*_*i*_ − min(*F*)}/{max(*F*) − min(*F*)}, where max(*F*) and min(*F*) are the maximum and minimum fluorescence value of all *F*_*i*_, respectively.

### Autocorrelation analysis

The principle approach used in this work is as follows: Using either raw fluorescence values *F*_*i*_ at a selected cycle number *i*, or *C*_*q*_ values estimated at a defined fluorescence threshold level *F*_Cq_ (Fig. 2A), we fit a quadratic model of the form 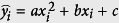 to the data (Fig. 2B). The rationale behind this approach is our observation of curvature and slope in *F*_*i*_ and *C*_*q*_ values with statistically significant quadratic coefficients. A Loess smoother with a span of 0.1 is then employed on the residuals *R*_*i*_ = *y*_*i*_ − 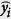 of the fit for the single purpose of an initial visualization of periodic patterns (Fig. 2C). A Wald-Wolfowitz (Runs) test and a Ljung-Box test are applied to the residuals in order to estimate significance of non-randomness and autocorrelation, respectively. We then use the residuals *R*_1_, *R*_2_, …. *R*_*i*_ from the fit for calculating the autocorrelation *r*_*k*_ with lags *k* = 1, 2, *…. n* by the following formula:


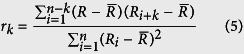


In a final step (Fig. 2D), we create the correlogram of all autocorrelations *r*_*k*_and use the automatic peak detection *R* procedure *findpeaks* to identify the period. The complete pipeline is implemented in the *CheckPeriod* function of Supplemental File 2.

### Estimation of other curve parameters

*C*_*q*_ values based on a defined threshold fluorescence *F*_*t*_, in the following termed *C*_*t*_, were estimated by inverse functions of the sigmoidal models. *C*_*q*_ values based on second-derivative maxima (*C*_*q*SDM_) were calculated by finding the cycle corresponding to the maximum value of the second-derivative function. In case of fitting with cubic splines, the root of the inverse or third-derivative of the spline function was employed to estimate *C*_*t*_ or *C*_*q*SDM_, respectively.

The maximum fluorescence *F*_max_ of a curve (“plateau phase”) was based on parameter *d* (upper asymptote) of a five-parameter sigmoidal model^8^.

### Computational aspects and reproducibility

All analyses in this work were conducted with the *R* statistical programming environment (www.r-project.org). The *qpcR* package^24^ was used for qPCR curve fitting and parameter estimation. To comply with the increasing need for computational reproducibility^25^, we provide data in Supplemental File 1 and the *R* script/workspace in Supplemental File 2, from which readers can reproduce all our figures.

### Web-based analysis of periodicity in qPCR data

A web-based analysis platform for investigating qPCR periodicity was developed with the Shiny framework for *R*^25^. Here the user can upload her/his qPCR data, either fluorescence values at a defined cycle or *C*_*q*_ values, and analyse the data with the pipeline given in Fig. 2. The web application is to be found at www.smorfland.uni.wroc.pl/shiny/period_app/. Overview screenshots of this application are given in Supplemental Fig. 5.

## Additional Information

**How to cite this article**: Spiess, A.-N. *et al*. System-specific periodicity in quantitative real-time polymerase chain reaction data questions threshold-based quantitation. *Sci. Rep.* **6**, 38951; doi: 10.1038/srep38951 (2016).

**Publisher’s note:** Springer Nature remains neutral with regard to jurisdictional claims in published maps and institutional affiliations.

## Supplementary Material

Supplementary Dataset 1

Supplementary Dataset 3

Supplementary Dataset 4

Supplementary Analysis and Figures

Supplementary Information
