# Supplementary material for: System-specific periodicity in quantitative real-time polymerase chain reaction data questions threshold-based quantitation
Source: Sci Rep. 2016 Dec 13;6:38951. doi: 10.1038/srep38951 (PMC5154181; doi:10.1038/srep38951)

# System-specific periodicity in quantitative real-time polymerase chain reaction data questions threshold-based quantitation

Andrej-Nikolai Spiess^1,*^, Stefan Rödiger^2,*^, Michał Burdukiewicz^3^, Thomas Volksdorf^4^ and Joel Tellinghuisen^5,a^

^1^ Department of Andrology, University Hospital Hamburg-Eppendorf, Hamburg, Germany

^2^ Faculty of Natural Sciences, BTU Cottbus - Senftenberg, Senftenberg, Germany

^3^ Department of Genomics, Faculty of Biotechnology, University of Wroclaw, Wrocław, Poland

^4^ Clinic and Polyclinic for Dermatology and Venerology, University Hospital Hamburg-Eppendorf, Hamburg, Germany

^5^ Department of Chemistry, Vanderbilt University, Nashville, Tennessee, USA 37235

**Supplementary raw data files without figures:**

**Supplemental File 1. Excel file with raw fluorescence qPCR data from six large technical replicate datasets.** These qPCR data were acquired with six different hardware systems, two different DNA-intercalating fluorescence dyes and four different amplicons.

**Supplemental File 2. *R* script and workspace for all analyses and figures.**

*R* script and workspace that was used to analyze the different technical replicate datasets as well as for creating all figures in this work.

**Supplemental File 3. Excel file with *C_q_*, *E* and *F*_0_ values obtained from the different qPCR quantitation methods as described in the Supplemental Data to Ruijter et al.^18^.** Samples that failed to be quantified by the original methods were imputed by spline interpolation (*R* function ‘na.spline’) as to enable autocorrelation analysis and are marked in red.

**Supplemental File 4. Fluorescence data for SybrGreen, ROX and Cy5 obtained from a non-template setup on a CFX96 (BioRad).**

40 cycles were run on qPCR setup in which one large mastermix (Maxima SYBR Green, Thermofisher) without template DNA was supplemented with the passive dye ROX and 150µM of an Oligo-dT_20_-Cy5 oligonucleotide. Fluorescence values for all three channels (green, orange-red, dark red) were acquired at each cycle. The mastermix was deposited in the wells of a 96-well microtiter plate with a single-channel pipettor.

**Supplemental Figure 1. Analysis of periodicity in *C_q_*, *E* and *F*_0_ values as obtained from the different qPCR analysis methods in Ruijter et al.^18^.**

**(A)** Autocorrelation analysis for *C_q_* values acquired by the six different methods shows strong periodicity for LinRegPCR, FPKM, DART and FPLM. **(B)** Same as in (A) but for estimated efficiencies *E*. None of the methods delivered periodic efficiencies. **(C)** Same as in (A) but for estimated target quantities *F*_0_. Strong periodicities are evident in LinRegPCR and FPLM. **(D)** The 'mak2' model delivers periodic *F*_0_ values. **(E)** The 'Cy0' method results in non-periodic *Cy*_0_ values. All methods displaying periodicity in autocorrelation analysis and non-randomness are marked with red boxes. X-axes denote lag number and y-axes denote autocorrelations. **(F)** Analysis of *C_q_* value dispersion for the different methods. Methods based on SDM (Cy0, Miner, 5PSM) exhibit significantly lower dispersion (narrower boxplot boxes, in blue) and lower coefficients of variation (C.V., in blue).

**
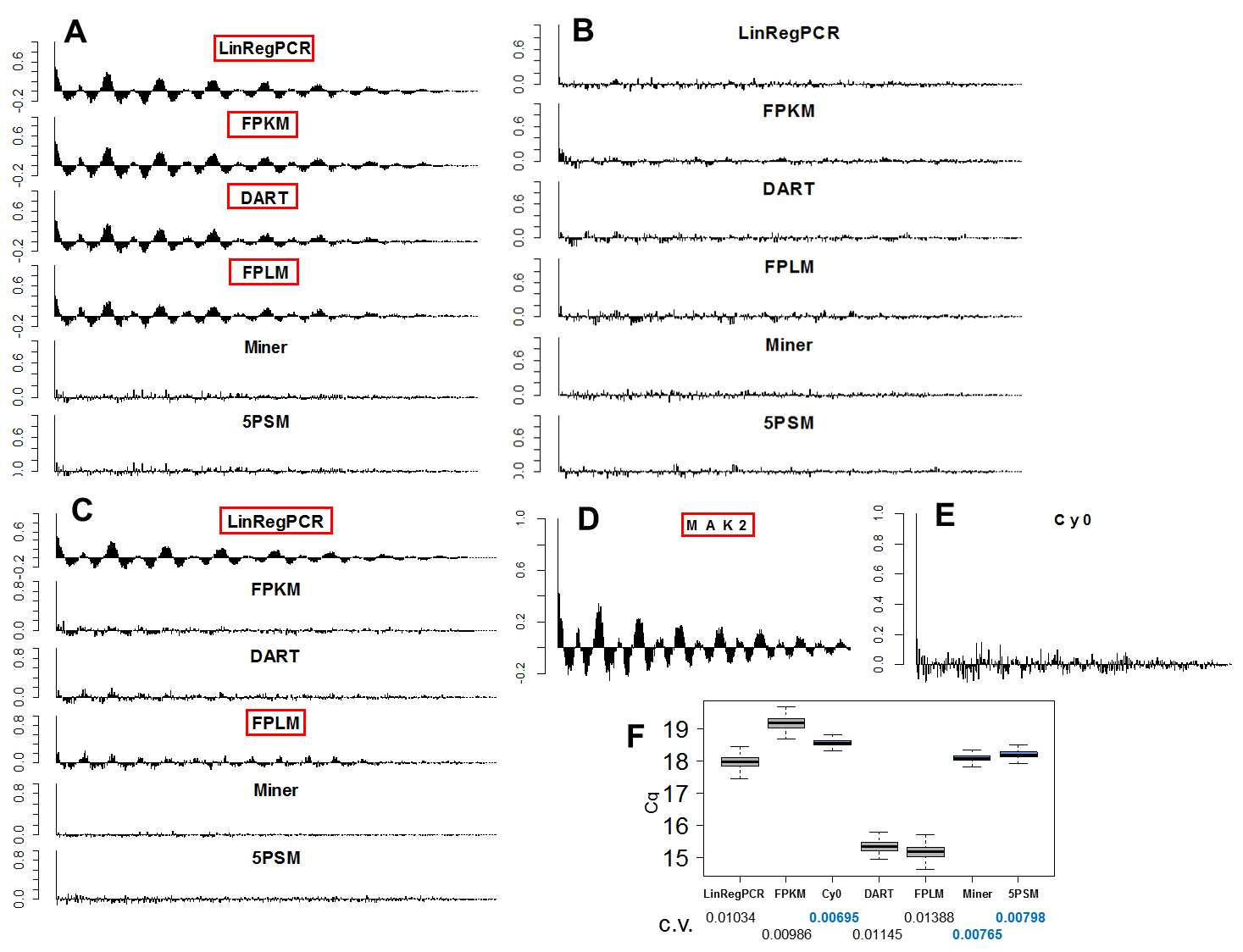
**

**Supplemental Figure 2. Periodicity analysis of *C_q_* values obtained from the CFX Manager™ software of the CFX96 real-time PCR system (Biorad).**

**(A)** Periodicity analysis of *C_q_* values obtained from the manual threshold method and **(B)** nonlinear regression method. The *C_q_* values were calculated from the ‘VIM.CFX96’ dataset of Supplemental File 1. Cq: *C_q_* value; RV: residual value; COR: autocorrelation.

**
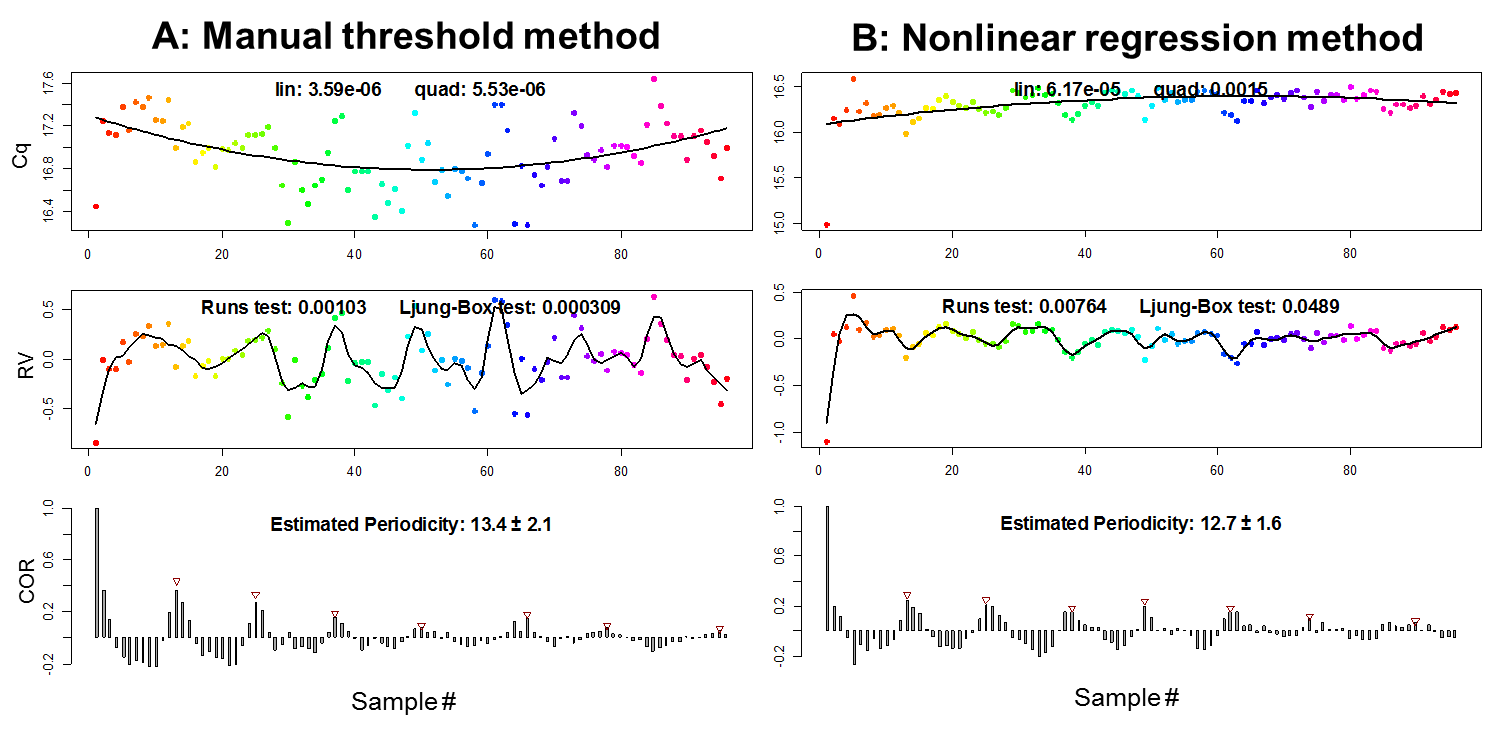
**

**Supplemental Figure 3. Periodicity analysis of *C_t_* values calculated from a published^34^ seven 10-fold dilution qPCR dataset with 12 replicates, obtained with a Lightcycler 480 instrument (Roche).**

**(A)** Raw fluorescence qPCR data was fit with a five-parameter sigmoidal model^8^ using the ‘qPCR’ package^24^ and *C_t_* values estimated at *F_t_* = 5. **(B)** Obtained *C_t_* values display the typical pattern of seven 10-fold dilutions with 12 replicates each (ΔCt ~ 3.3). **(C)** Plot of *C_t_* values after rescaling each replicate group into the interval [0, 1]. A periodic pattern is already visible. **(D)** The autocorrelation plot of rescaled *C_t_* values uncovers a strong periodic pattern with a period of 12. Ct: *C_t_* value; COR: autocorrelation.

**
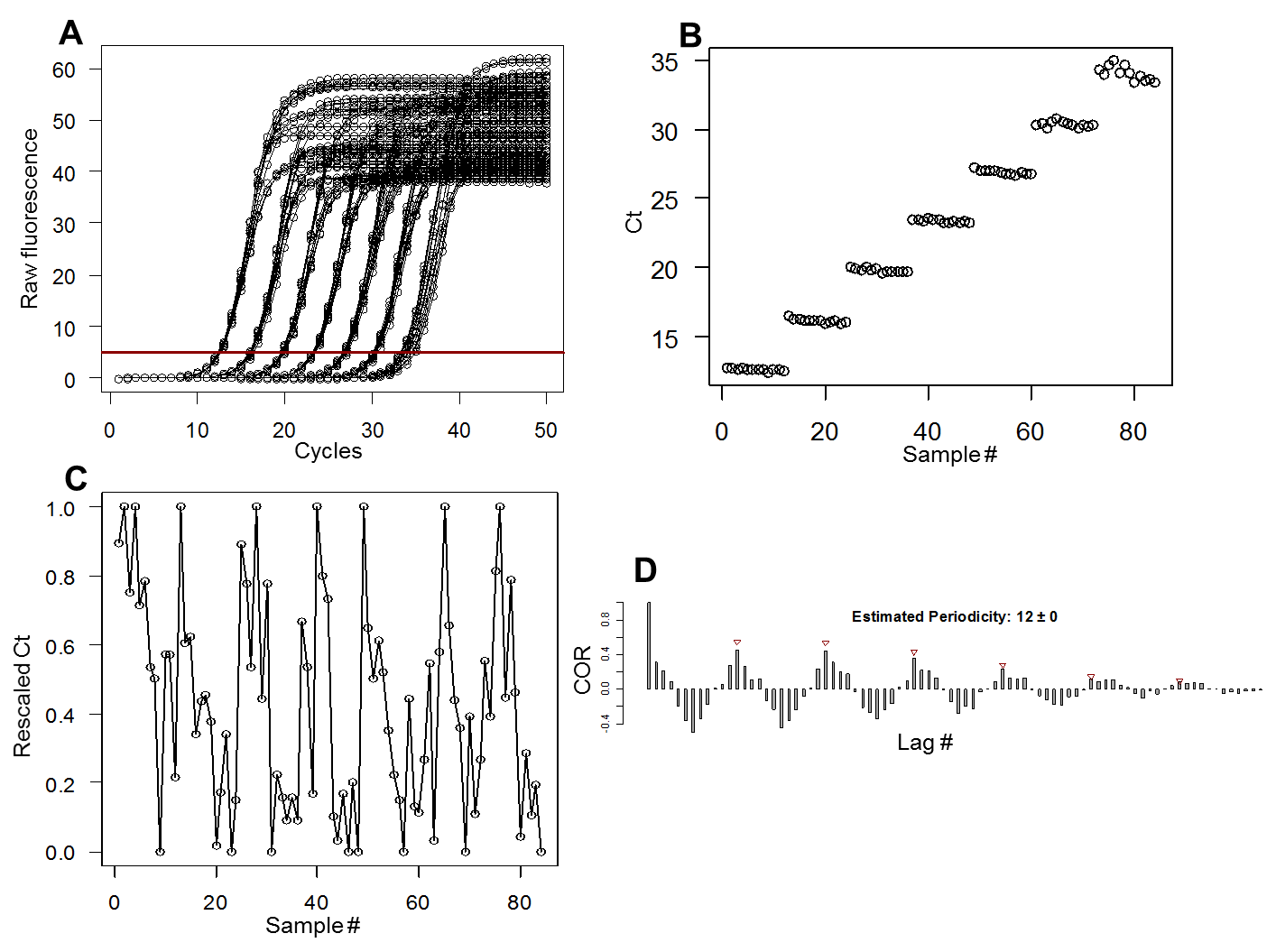
**

**Supplemental Figure 4. Effect of extreme *C_t_* values on calibration curve-derived efficiencies and copy numbers from unknowns.**

**(A)** Periodicity analysis of *C_t_* values obtained from the manual threshold method at *F_t_* = 500 for the first 94 samples (lowest dilution, 15000 copies) of the ’94-replicates-4-dilutions’ dataset in Ruijter et al.^18^. *C_t_* values were acquired by fitting a spline model to the baselined (average of first 8 cycles) raw data. Strong periodicity is evident as in the ‘380-replicates set’^18^ because both datasets were obtained with the same hardware (BioRad CFX384). **(B)** Generation of 34968 calibration curves using all 4-point combinations of the two extreme (minimum and maximum) *C_t_* values of the lowest (15000 copies) and highest (15 copies) dilutions and all 94 *C_t_* values of the two intermediate dilutions (1500 and 150 copies). *C_t_* values were plotted against log_10_(*N*) and a linear model fitted to each of the combinations. **(C)** Boxplot of the 34968 qPCR efficiencies calculated from the regression slopes of (B) by 10^-slope^. Efficiencies spread within a window of 1.79 to 2.19. **(D)** Boxplot of 34968 copy number estimations derived at *C_t_* = 30 (blue vertical line in (B)). Copy numbers spread within a window of 28 to 86 copies. COR: autocorrelation.

**
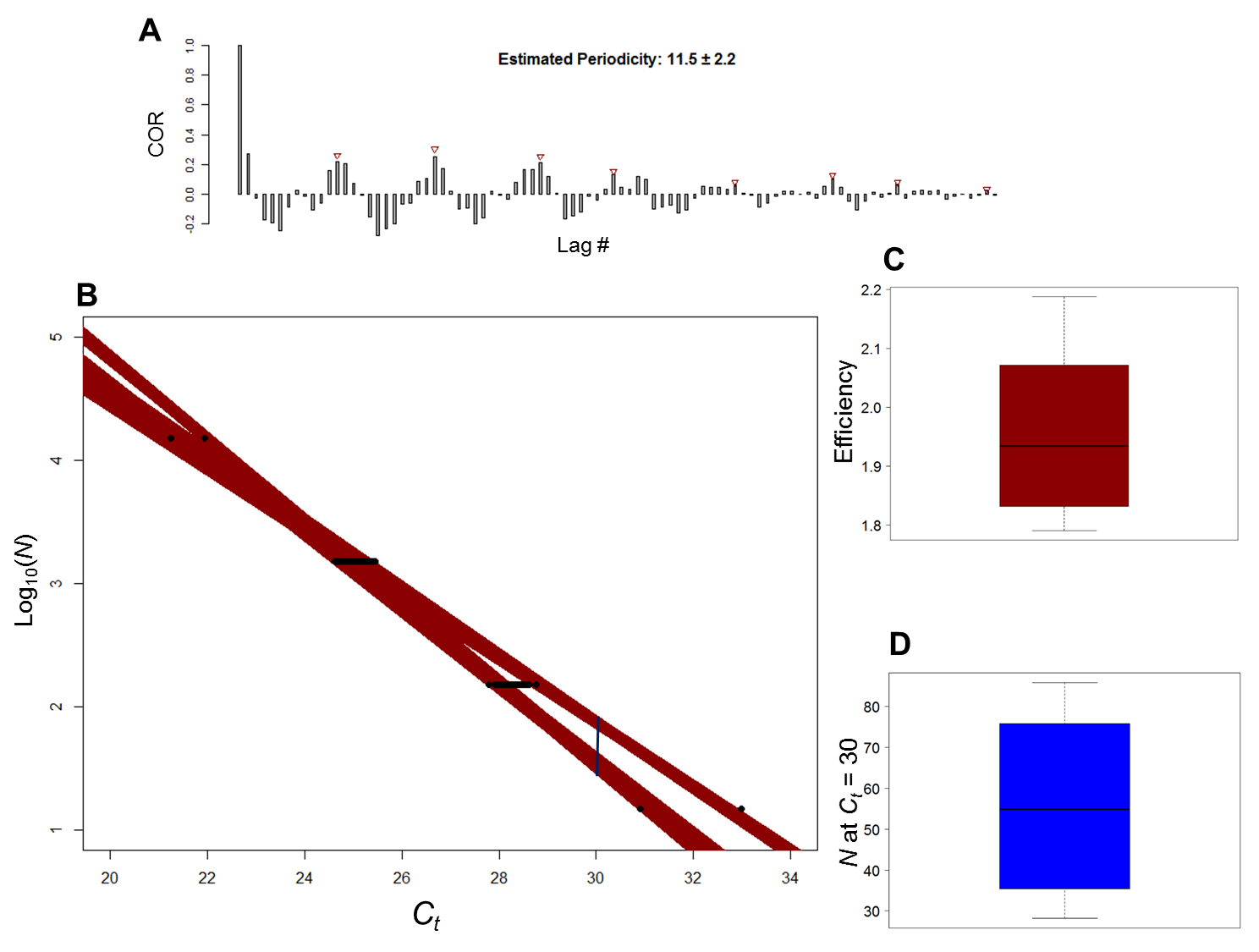
**

**Supplemental Figure 5. Screenshots of our web application for periodicity analysis in qPCR data**.

**(A)** The user is instructed on how to load the input data into the web application. Options include the selection of the decimal separator. Example data, describing the data structure, are available. **(B)** The web application’s tabs offer a graphical output ("Results with graphics") of the analysis and a tabular view of the input data ("Input table"). **(C)** The autocorrelation analysis is presented with automatic peak estimation (red triangles). **(D)** Residuals from the quadratic fit are mapped to their corresponding plate positions.


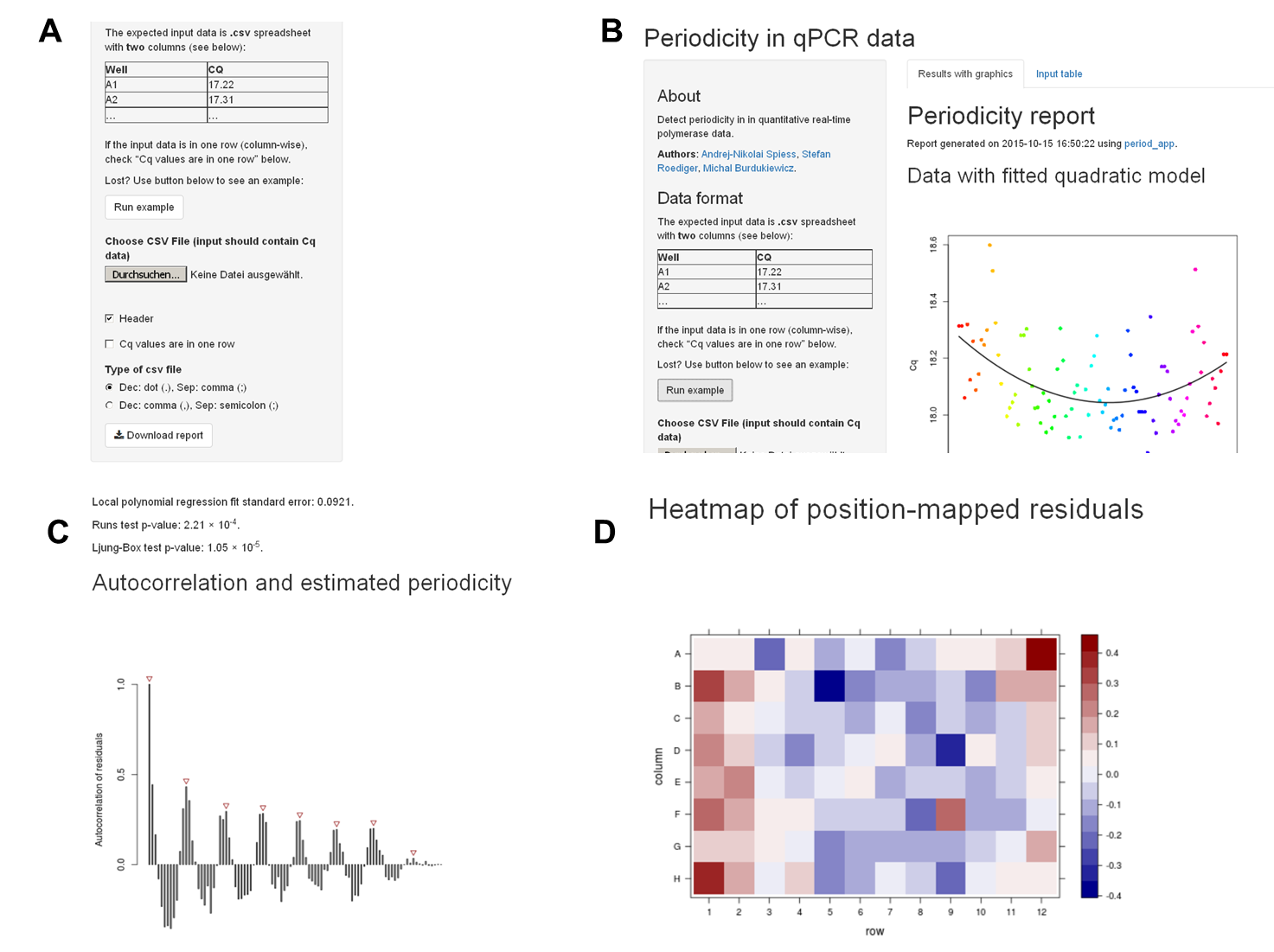

Supplement: Supplementary Information [file srep38951-s5.doc]
